# Unsteady Magnetohydrodynamic Free Convection Flow of a Second Grade Fluid in a Porous Medium with Ramped Wall Temperature

**DOI:** 10.1371/journal.pone.0088766

**Published:** 2014-05-01

**Authors:** Samiulhaq, Sohail Ahmad, Dumitru Vieru, Ilyas Khan, Sharidan Shafie

**Affiliations:** 1 Department of Mathematics, City University of Science and Information Technology, Peshawar, Pakistan; 2 COMSATS Institute of Information Technology, Attock, Pakistan; 3 Technical University “Gheorghe Asachi” of Iasi, Iasi, Romania; 4 College of Engineering Majmaah University, Majmaah, Saudi Arabia; 5 Department of Mathematical Sciences, Faculty of Science, Universiti Teknologi Malaysia, Skudai, Johor, Malaysia; University of Adelaide, Australia

## Abstract

Magnetic field influence on unsteady free convection flow of a second grade fluid near an infinite vertical flat plate with ramped wall temperature embedded in a porous medium is studied. It has been observed that magnitude of velocity as well as skin friction in case of ramped temperature is quite less than the isothermal temperature. Some special cases namely: (i) second grade fluid in the absence of magnetic field and porous medium and (ii) Newtonian fluid in the presence of magnetic field and porous medium, performing the same motion are obtained. Finally, the influence of various parameters is graphically shown.

## Introduction

The natural convection heat transfer from a vertical plate to a fluid has applications in many industrial processes. It was extensively studied by a number of researchers using different sets of thermal conditions at the bounding plate. Special mention can be made, for instance, to the studies of Raptis and Sing [1], [2], Sacheti *et al.* [3], Chandran *et al.* [4], [5] and Ganesan and Palani [6] that have determined analytical solutions for velocity and temperature using continuous and well-defined conditions at the wall. Samiulhaq *et al.* [7] discussed the influence of radiation and porosity on the unsteady magnetohydrodynamic (MHD) flow past an infinite vertical oscillating plate with uniform heat flux in a porous medium. Keeping in mind the importance of shear stress on the boundary, Fetecau *et al.* [8] reinvestigated the problem of Samiulhaq *et al.* [7] by considering shear stress on the boundary. However, some practical problems may require non-uniform or arbitrary wall conditions. Chandran *et al.* [9] studied the unsteady free convection flow of an incompressible viscous fluid near a vertical plate with ramped wall temperature and compared the results with those of the plate with constant temperature. Recently, Seth and Ansari [10] and Seth *et al.* [11] found exact solutions for the MHD natural convection flow past an impulsively moving vertical plate with ramped wall temperature in the presence of thermal diffusion or radiation heat transfer. Narahari and Beg [12] considered the problem of Chandran *et al.* [9] for the impulsive motion of the plate with radiation and constant mass diffusion. More recently, Samiulhaq *et al.* [13] investigated the unsteady MHD flow past an impulsively started vertical plate present in a porous medium with thermal diffusion and ramped wall temperature. However, all aforementioned results refer to incompressible viscous fluids.

Due to increasing significance of non-Newtonian fluids over the past few years, several researchers in the field are involved by valuable contributions in the study of flows of non-Newtonian fluids. It is due to their numerous applications in several areas, such as the plastic manufacture, performance of lubricants, food processing, or movement of biological fluids. These fluids are defined by a non-linear constitutive relationship between the stress and the rate of deformation tensors and, therefore, various models of non-Newtonian fluids have been proposed. Amongst them, the second grade fluids are the simplest subclass for which one can easily obtain analytical solutions. For these reasons and because, the second grade fluids can model many fluids such as dilute polymer solutions, slurry flows, industrial oils, many flow problems with various geometries and different mechanical and thermal boundary conditions have been studied.

Tan and Masouka [14] investigated the Stokes’ first problem for a second grade fluid in a porous half-space with a heated flat plate. Hayat and Abbas [15], by means of homotopy analysis method, have studied the heat transfer on the MHD flow of second grade fluids in a channel with porous medium. Closed form solutions for MHD flow of a second grade fluid through porous space are obtained by Khan et al. [16]. Thermal effects in Stokes’ second problem for second grade fluid through a porous medium under the effect of magnetic field have been investigated by Srinivasa Rao et al [17]. Mustafa et al [18] have studied free convection flow of a viscoelastic second grade fluid along a vertical plate with power- law surface temperature.

The influence of magnetic field is observed in several natural and human-made flows. Magnetic fields are commonly applied in industry to pump, heat, levitate and stir liquid metals. There is the terrestrial magnetic field which is maintained by fluid flow in the earth’s core, the solar magnetic field which originates sunspots and solar flares, and the galactic magnetic field which is thought to control the configuration of stars from interstellar clouds [19]. Three major technological innovations namely, (i) fast-breeder reactors used liquid sodium as a coolant which requires pumping; (ii) controlled thermonuclear fusion needs that the hot plasma be confined away from material surfaces by magnetic forces; and (iii) MHD power generation, in which ionized gas is propelled through a magnetic field were made by incorporating MHD in the field of engineering. The phenomenon concerning heat and mass transfer with MHD flow is important due to its numerous applications in science and technology Hayat *et al.* [20], [21] and Hayat and Qasim [22]. The particular applications are found in buoyancy induced flows in the atmosphere, in bodies of water and quasi-solid bodies such as earth. Therefore, heat and mass transfer with MHD flow has been a subject of concern of several researchers (see for example, Katagiri [23], Jana et al. [24], Mandal and Mandal [25], Gosh [26], Jha and Apere [27], and the references therein). Other interesting results regarding the second grade fluids can be found in the references [28]–[34].

The purpose of this note is to extend some of the previous results to a larger class of fluids, namely to second grade fluids. More exactly, we establish exact solutions for velocity and temperature corresponding to the natural convection flow of a second grade fluid near an infinite vertical plate with ramped wall temperature. Apart from several other applications, the present study is significant and worthwhile as the exact solutions obtained in this paper are important not only that these solutions are new but as they can be used as checks for many approximate solutions and as tests for verifying numerical schemes. These solutions, obtained both for 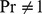 and 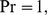
satisfy all imposed initial and boundary conditions. For comparison, the solutions corresponding to the plate with constant temperature are also established. Finally, temporal and spatial variations of velocity as well as those of the wall skin friction are graphically discussed.

## Mathematical Formulation of the Problem

**Figure 1:**
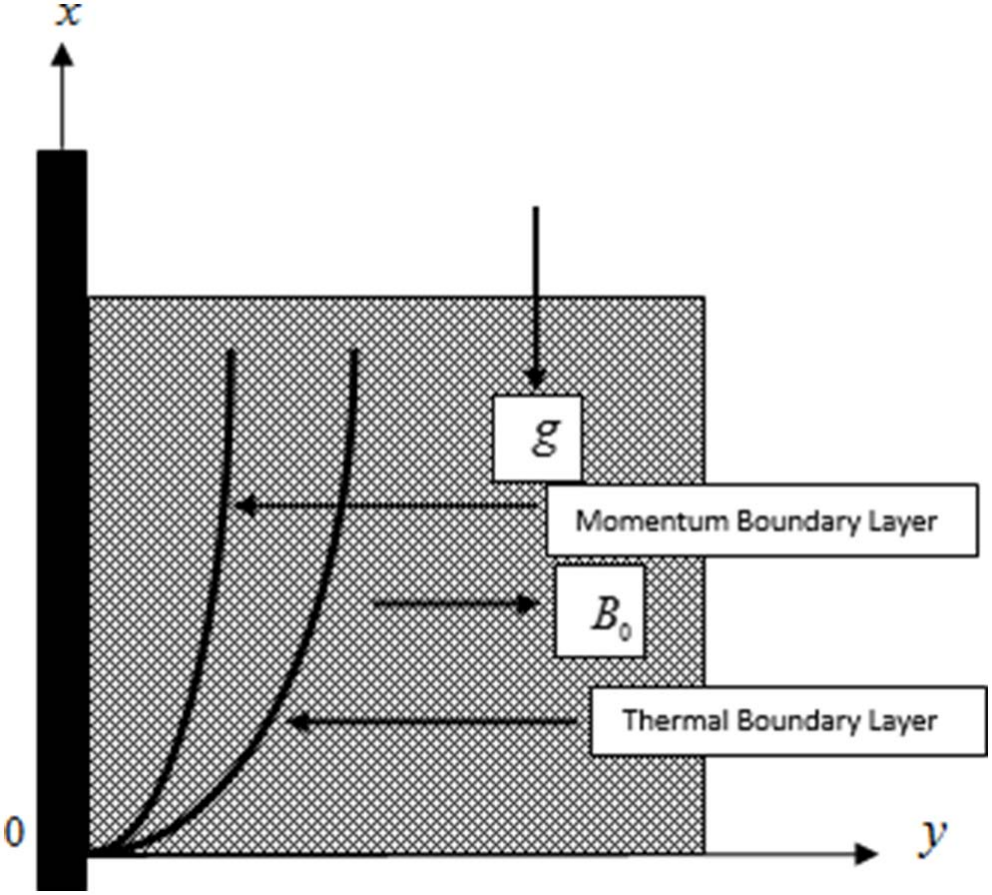
Physical system and coordinate axes.

Let us consider the unsteady MHD flow of an incompressible second grade fluid near an infinite vertical plate with ramped wall temperature. The flow of electrically conducting fluid is taken in a porous medium. The 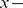
axis is taken along the plate in the upward direction and 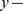
axis is taken normal to the plane of the plate. A uniform magnetic field of strength 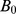 is acting in transverse direction to the flow as shown in Figure 1. Initially, at time 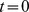, both the fluid and the plate are at rest to a constant temperature 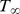. At time 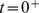, the temperature of the plate is raised or lowered to 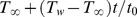 when 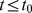, and thereafter, for 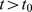, is maintained at the constant temperature 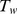. The main purpose here is to study the free convection flow resulting from the ramped temperature profile of the bounding plate.

It is assumed that the effects of viscous dissipation are negligible in the energy equation. One of the body force term corresponding to an MHD flow is the Lorentz force 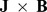. Where 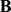 is the total magnetic field and 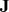 is the current density. By using Ohm’s law, the current density is given as
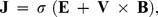
(1)where 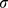 is electrical conductivity of the fluid, 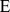 is the electric field, 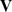 is the velocity vector field, 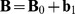
with 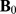 is the imposed magnetic field and 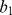
is the induced magnetic field. The current density 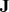 with the assumptions 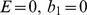 and 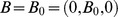, where 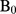 is the strength of applied magnetic field 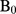, modifies to 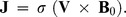 Finally the Lorentz force becomes
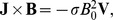
as mentioned by Hayat *et al.* [28]. For the problem under consideration, we assume the velocity of the following form 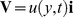 where 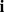 is unit vector along 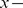
axis. Under the usual Boussinesq’s approximation of temperature gradient the equations governing the flow are:



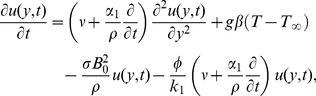
(2)

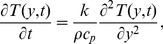
(3)Here 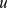
is the velocity of the fluid in the 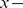
direction, 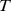
is its temperature, 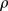 is the density, 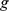
is the acceleration due to gravity, 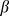 is the volumetric coefficient of thermal expansion, 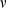 is the kinematic viscosity, 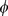 is the porosity of the porous medium, 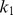 is the permeability, 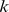 is the thermal conductivity, 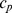 is the specific heat of the fluid at constant pressure, and 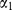 is one of the material module of second grade fluids.

The initial and boundary conditions are:
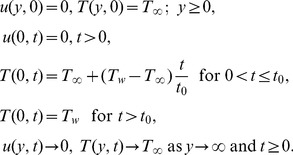
(4)


Introducing the following non-dimensional physical quantities
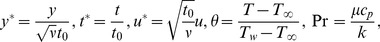
(5)


into Eqs. (2) and (3) and dropping out the “*” notation we get
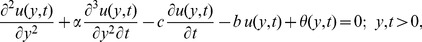
(6)

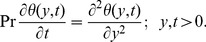
(7)


The adequate initial and boundary conditions are



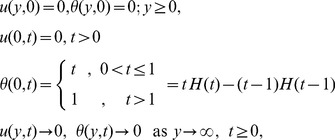
(8)where 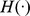
is the Heaviside step function and
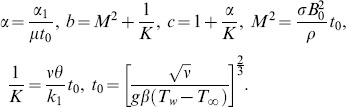



## Solution of the Problem

In the following, exact analytical solutions for the coupled partial differential equations (6) and (7) with the initial and boundary conditions (8) will be determined by means of Laplace transforms. For comparison, the solutions corresponding to an isothermal plate with constant temperature are also established. Applying the Laplace transform to Eqs. (6), (7) and (8), we obtain the transformed equations
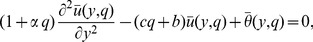
(9)

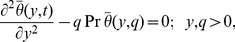
(10)where 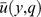
and 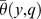
are Laplace transforms of 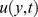
and 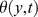, together with the initial and boundary conditions in the transformed domain



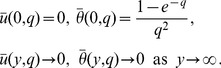
(11)The equation (10) is uncoupled to Eq. (9) and its solution with the corresponding conditions (11) is
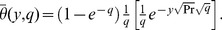
(12)


Denoting by
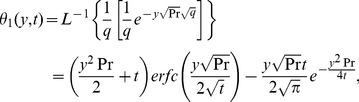
(13)


and using the second shift property
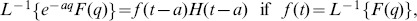



we obtain the following known result for the temperature distribution [9, Eq. (11)]
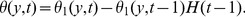
(14)


The solution corresponding to Eqs. (9), (11)_1_ and (11)_3_ is given by
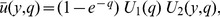
(15)where
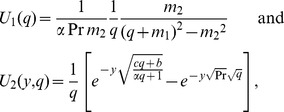
(16)with 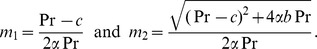
The inverse Laplace transform 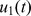 of 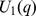
is given by,



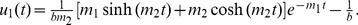
(17)In order to determine the inverse Laplace transform 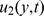 of the function 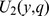, we consider the following function:
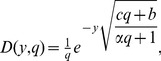
(18)whose inverse Laplace transform is given by
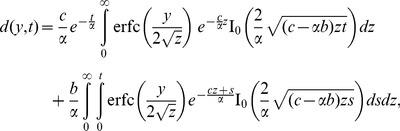
(19)and get
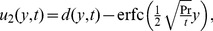
(20)where 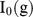 is modified Bessel function of the first kind of order zero and 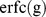
is complementary error function. Consequently, the expression for velocity in the 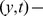
domain, can be written in the simple form
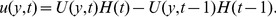
(21)where
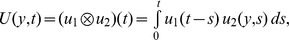
where the symbol 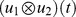
denotes convolution of 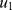
and 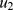. A simple analysis clearly shows that both solutions (14) and (21), satisfy all imposed initial and boundary conditions. In order to show that 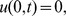 for instance, we need the following integrals.
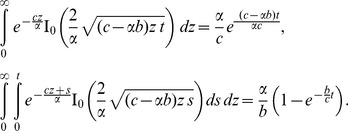



## Solutions for Plate with Constant Temperature

In order to bring to light the effects of ramped temperature of the plate on the fluid flow, we must compare our results with those corresponding to the flow near a plate with constant temperature. In this case, the initial and boundary conditions are the same excepting Eq. (8) that becomes 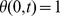
for 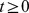. The expression for the dimensionless temperature 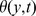 is again the same obtained by Chandran *et al.* [9, Eq. (19)], i.e.
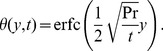
(22)


Introducing the expression of 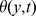 into Eq. (9), and following the same way as before we find that
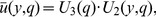
(23)where
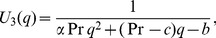
(24)and its inverse Laplace transform is



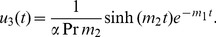
(25)Consequently, the dimensionless velocity corresponding to this case is
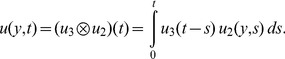
(26)


## Nusselt Number and Skin Friction

From velocity and temperature fields, the expressions for Nusselt number and skin friction can easily be determined. They are measures of the heat transfer rate and shear stress at the boundary. The Nusselt number Nu, can be written as
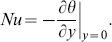
(27)


Introducing equations (14) and (22) into (27), we obtain:

The Nusselt number for ramped temperature
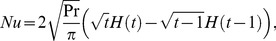
(28)and for isothermal temperature as



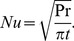
(29)As regards the skin friction, in dimensionless form, is
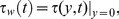
(30)where the shear stress 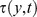
is given by [28]



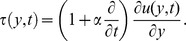



Using equations (21) and (26) into above equation, we obtain:

the shear stress for ramped temperature as
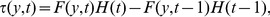
(31)where



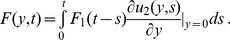



The shear stress for constant temperature is
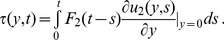
(32)where



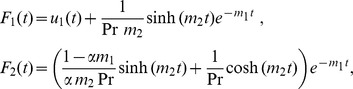
and





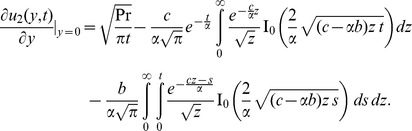



## Special Cases

Te solutions corresponding to the flow of a second grade fluid with ramped wall temperature or constant temperature on the boundary in the absence of magnetic or porous effects can be immediately obtained from the general solutions (21) and (26) by making 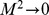 or 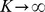, respectively. However, if 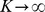, the constant 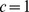
and the corresponding solutions are different for 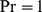
and 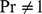. So, for completion, we also give the exact solutions for velocity in two special cases.

1. The case 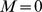 and 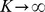

By making 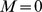
and 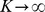, it results 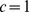
and 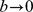. The function 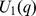 from Eq. (16)_1_ becomes
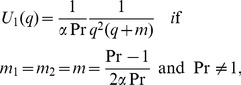
(33)or



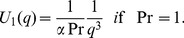
(34)The corresponding velocity 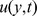, after lengthy but straightforward computations, is found to be
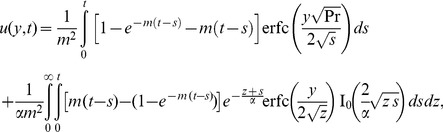
(35)for Pr≠1 respectively,

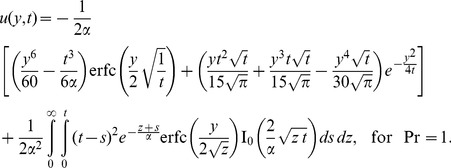
(36)2. The case 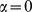 (Newtonian fluid with MHD and Porosity)

For 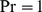


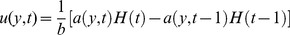
(37)where



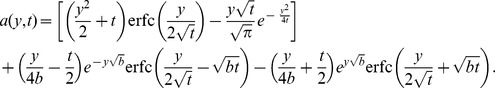


**Figure 2:**
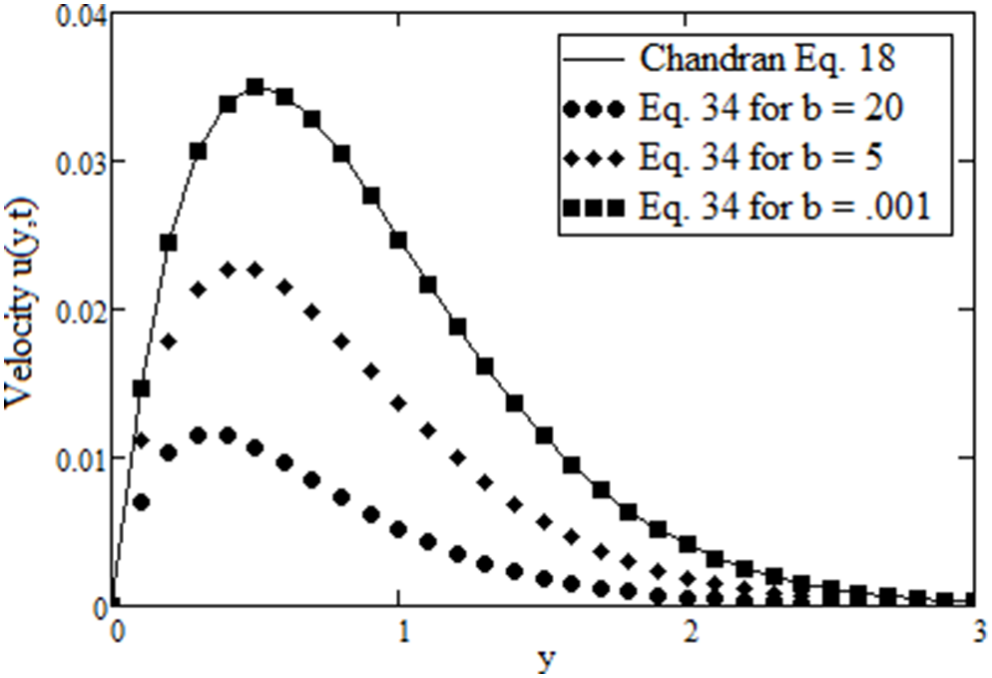
Comparison of velocity 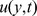 in Eq. (37) with Eq. (18) in Chandran *et al.* [9].

In Figure 2, by making 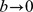
it is observed that the graph of 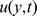
in Eq. (37) is similar to that of Eq. (18) from Chandran *et al.* [9] given by
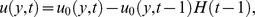
where 
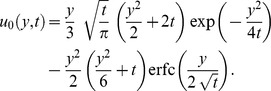


## Numerical Results and Discussion

The effects of different flow parameters have been analyzed by numerical calculations and graphical illustrations. A numerical algorithm was used in order to compare the analytical solutions with the numerical solutions.

**Figure 3:**
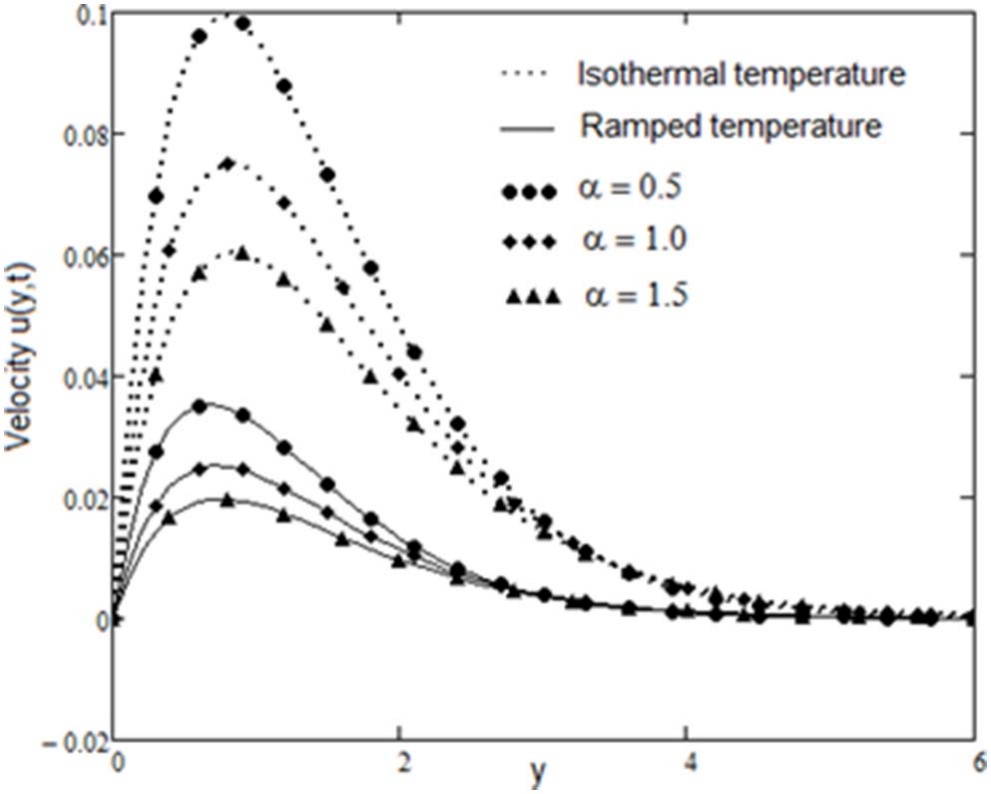
Velocity profiles for different values of 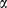 with 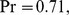 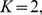 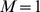 and 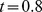.

**Figure 4:**
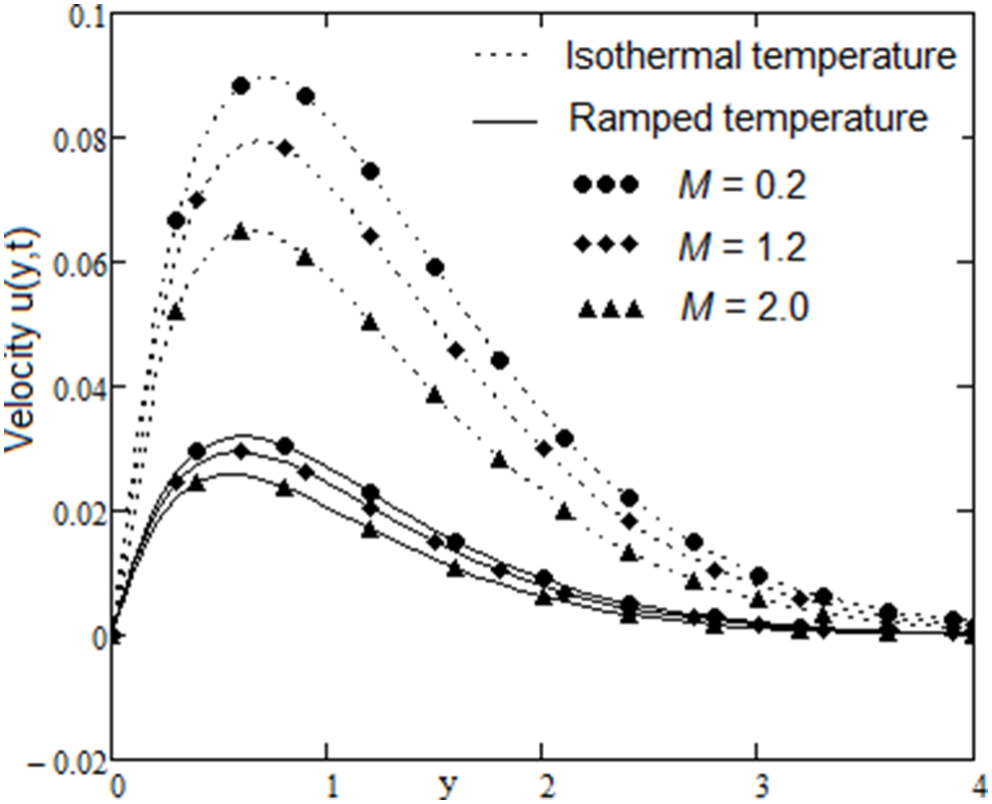
Velocity profiles for different values of 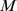 with 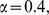 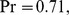 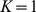 and 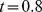.

**Figure 5:**
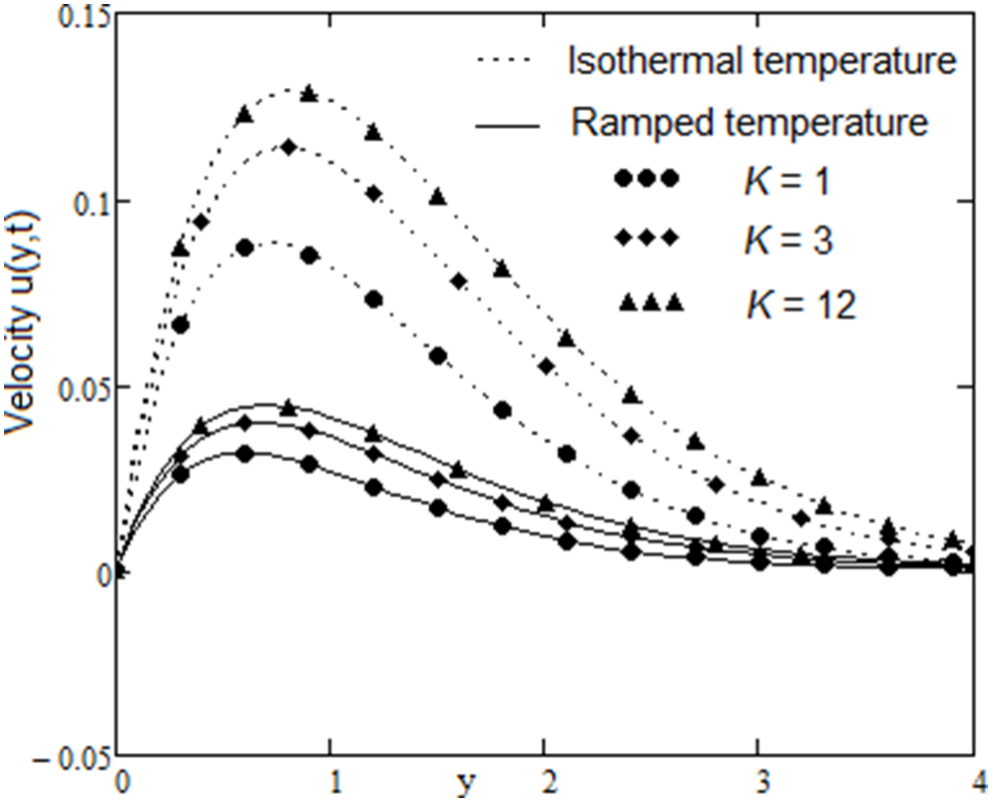
Velocity profiles for different values of 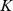 with 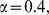 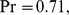 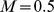 and 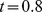.

**Figure 6:**
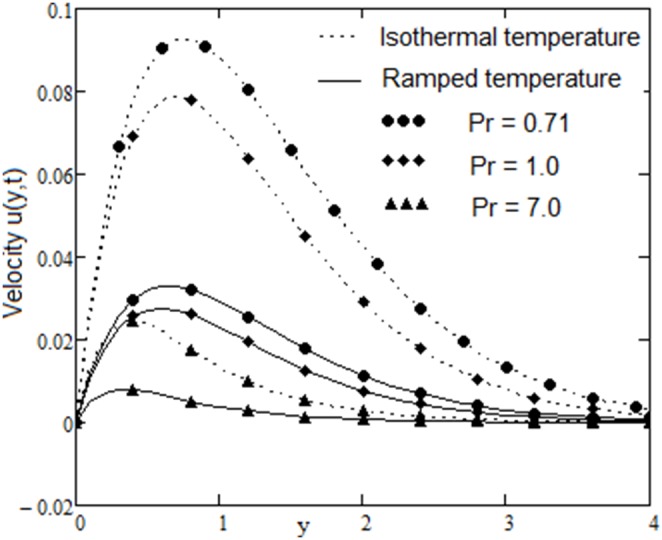
Velocity profiles for different values of 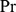 with 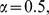 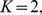 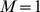 and 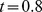.

**Figure 7:**
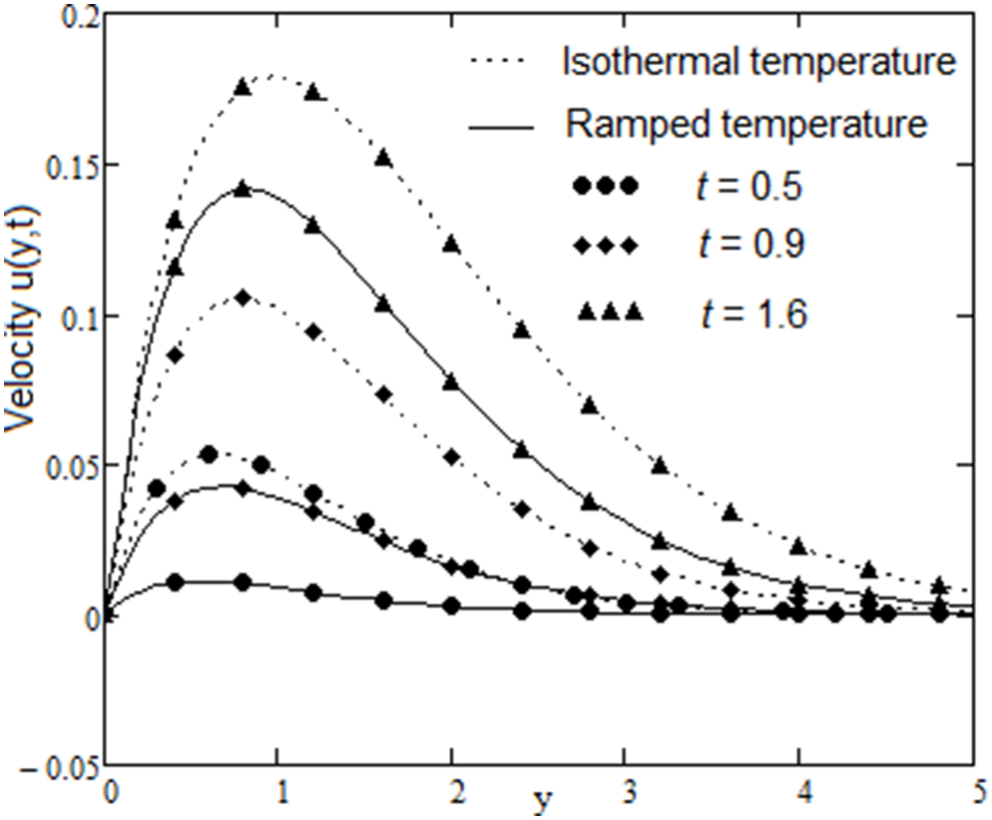
Velocity profiles for different values of 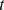 with 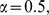 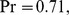 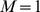 and 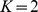.

**Figure 8:**
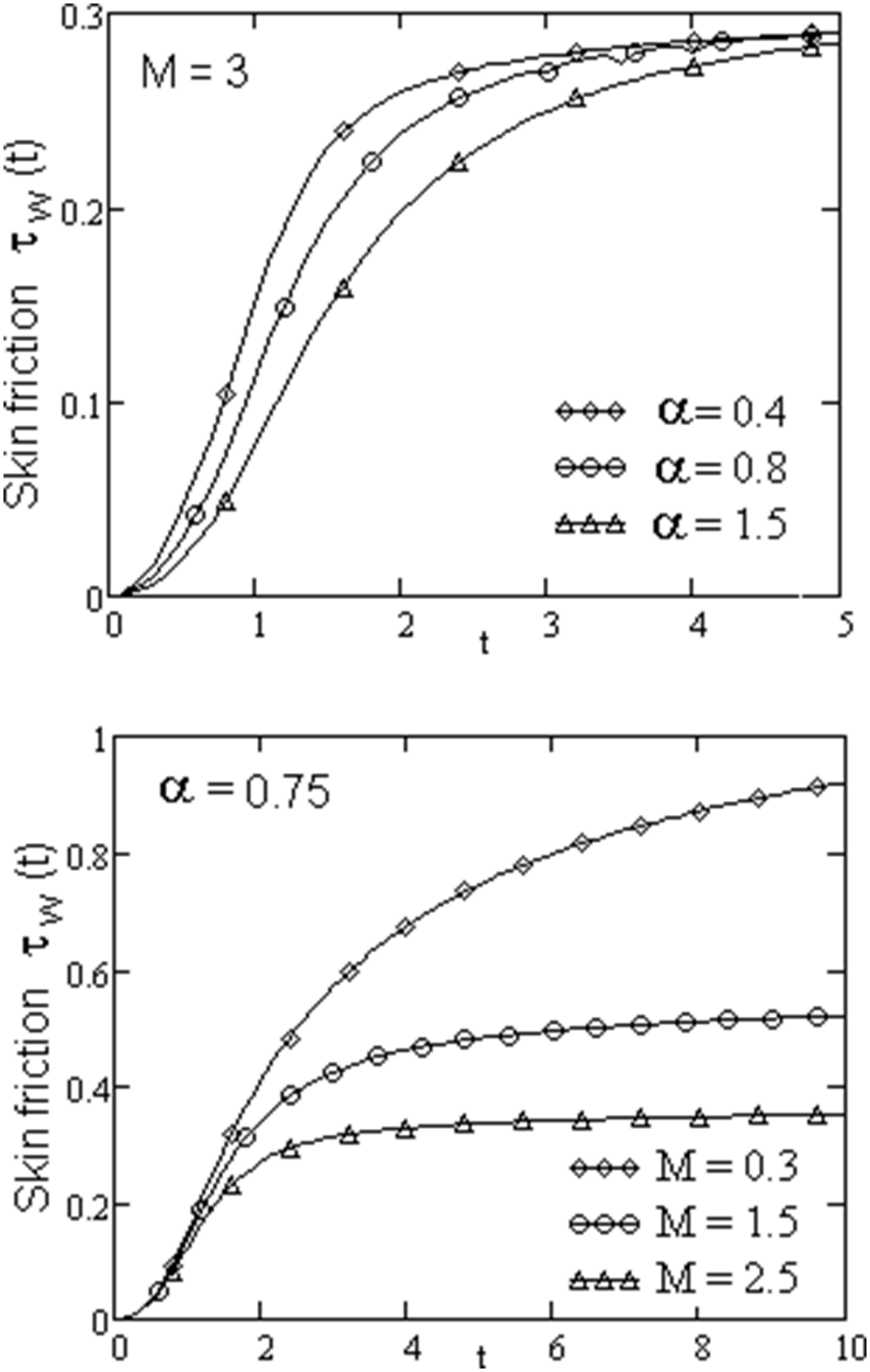
Variations of the skin friction for different values of 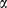 and 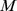 with 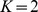 and 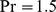.

The velocity field, for various values of second grade parameter α, is described in Fig. 3. The effect of the second grade parameter is to decrease velocity throughout the flow field when α increases. It is also clear that, the velocity approaches to zero at the far away from the plate. It is noticed that, the thickness of the boundary layer increases if the second grade parameter decreases. For ramped temperature on the plate, fluids flow slower than for the constant plate temperature. The effect of the magnetic strength on the motion of the fluid, for both heating cases, is analyzed in Fig. 4. Increasing of the magnetic parameter decelerates the motion of the fluid in the boundary layer. Therefore, the magnetic field acts like a drag force. The influence of the permeability parameter K is shown in Fig. 5. It is observed that the velocity field is an increasing function of K. As expected, the increase of the permeability of the porous medium reduces the drag force and, therefore, fluid velocity increases. The effect of Prandtl number on the velocity field is sketched in Fig. 6. It is also clear that, the increase of the Prandtl number decelerates the motion of the fluid. In Fig. 7 are plotted the diagrams of velocity u(y, t), versus t, for both cases of the plate heating. The fluid velocity is an increasing function of time t in the boundary layer then, finally it approaches to zero. In Fig. 8 are plotted the diagrams of the skin friction 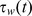 given by Eq. (31), for several values of the second grade parameter α and magnetic parameter M. If both parameters increase, then the skin friction decreases. For a short time-interval the skin friction increases then approaches to a constant value.

In order to obtain the closed form (21) of solution, we have used the Laplace transform method. In many problems, the inversion of image - functions can be a difficult problem. Even if in our work, the inversion of function (15) is not too difficult, we present a numerical technique for inversion, namely, the Stehfest’s algorithm [35]. Based on the Stehfest results, the inverse Laplace of the function H(y, q) is given by
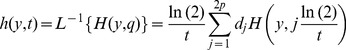
(38)where p is a positive integer and



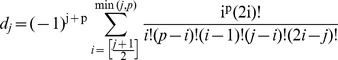
(39). Here [r] denotes the integer part of the real number r and



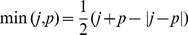
(40)


**Figure 9:**
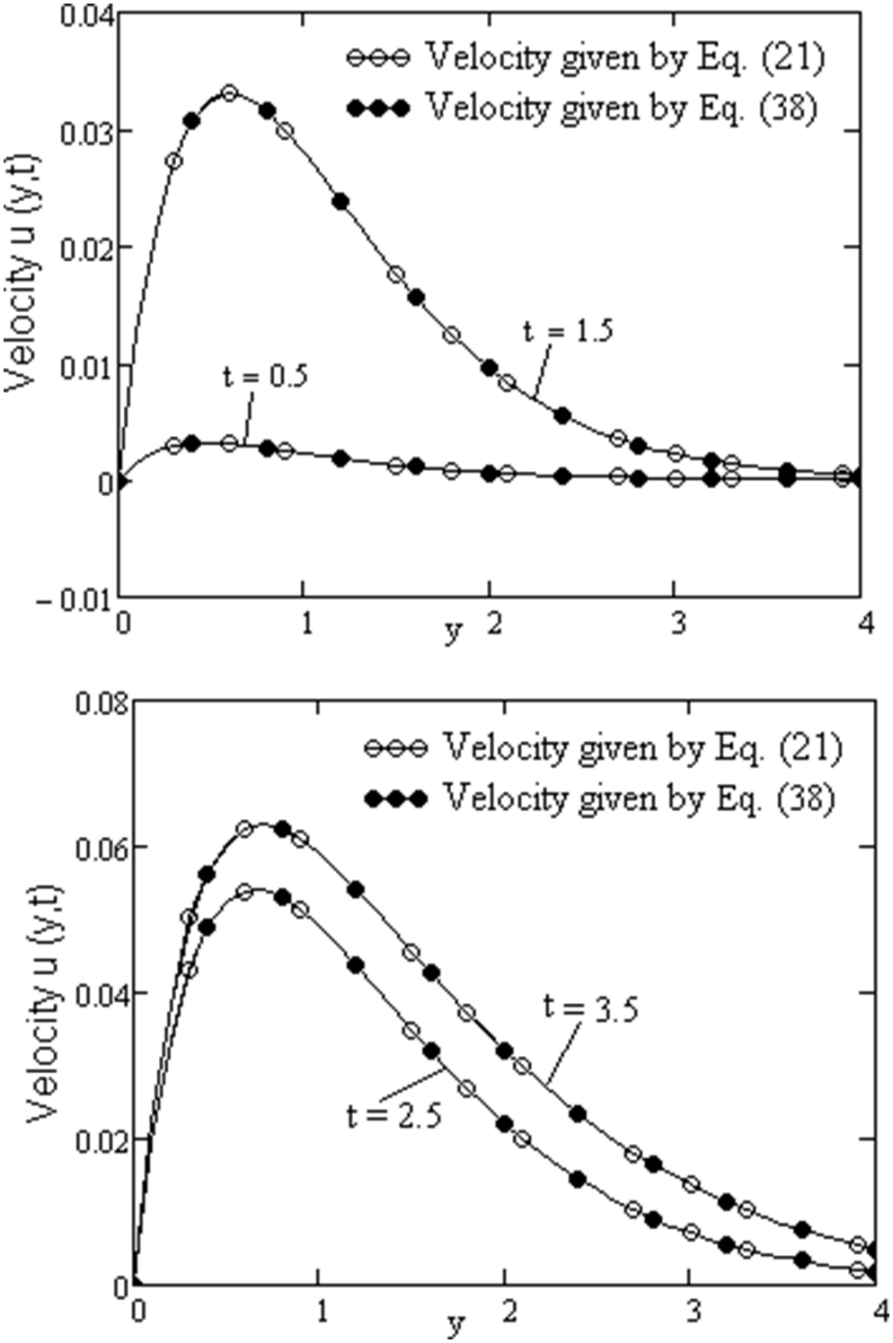
Comparison between values obtained with (21) and (38) for 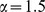, 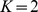, 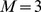, 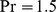.

Applying the formula (38) to Laplace transform 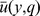 given by equation (15), the values of velocity field u(y, t) are obtained. As shown in Figure 9 and in Table 1, the values of the function u(y, t) obtained by formulae (21) and (38) are in excellent agreement. In Table 1 we denoted by u(y, t) and v(y, t) the values of velocity given by Eq. (21), respectively, by Eq. (38). The Table1 contains the absolute errors 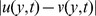. Figure 9 confirms the velocity properties shown in Figure 7, namely the increasing of velocity in the boundary layer when, the time increases.

**Table 1:** Absolute errors of velocity calculated by Eqs. (21) and (38).

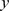	Absolute errors 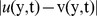			
	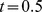	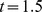	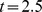	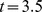
0	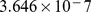	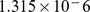	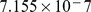	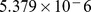
0.1	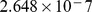	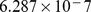	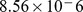	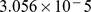
0.2	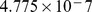	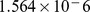	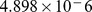	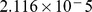
0.3	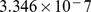	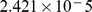	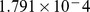	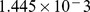
0.4	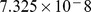	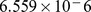	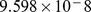	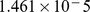
0.5	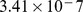	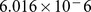	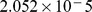	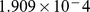
0.6	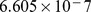	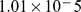	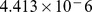	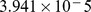
0.7	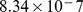	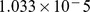	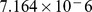	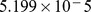
0.8	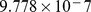	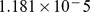	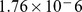	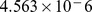
0.9	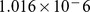	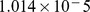	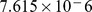	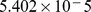
1.0	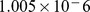	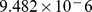	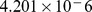	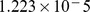
1.1	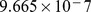	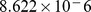	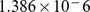	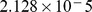
1.2	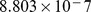	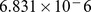	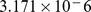	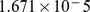
1.3	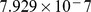	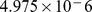	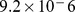	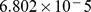
1.4	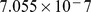	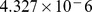	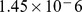	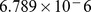
1.5	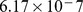	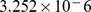	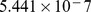	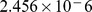
1.6	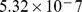	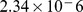	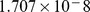	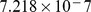
1.7	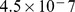	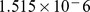	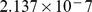	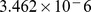
1.8	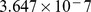	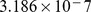	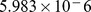	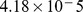
1.9	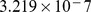	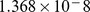	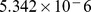	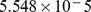
2.0	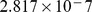	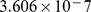	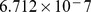	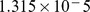

## Limitations of the Study and Future Recommendations

It is important to bring to light various limitations of this research. A discussion of these limitations will not only assist readers to understand this study, but also provide an opportunity to extend the current research. The following assumptions and limitations are considered

- Flow is incompressible and laminar.
- Flow is one dimensional and uni-directional.
- A uniform magnetic field is applied outward direction perpendicular to the flow.
- It is assumed that the effects of viscous dissipation in the energy equation are negligible.
- Electric field due to polarization of charges is not considered.

The mathematical model of second grade fluids offers, in general, possibilities to find of analytic solutions. Unfortunately, this model does not exhibit some significant features of some fluids. From this reason the present work can be extended to other more complex models, such as the power - law fluids of second grade in which the fluid may exhibit normal stresses, shear thinning or shear thickening behavior. Also, the approaches of some models with fractional derivatives in various geometrical configurations can be interesting. The present study provides analytical solutions in the closed form which can be used as a benchmark by numerical analysts.

## Conclusions

Exact solutions corresponding to the ramped wall temperature of unsteady MHD free convection flow of a second grade fluid in a porous medium are established. Solutions are obtained by using Laplace transform technique. The obtained solutions can easily be reduced to similar solutions for Newtonian fluids. They can be used to develop new exact solutions corresponding to free convection flows of several non-Newtonian fluids. The corresponding expressions for skin friction and Nusselt number are also obtained. Graphical results for velocity and skin friction are presented to understand the physical behavior of the involved flow parameters. Finally, the following observations are made from the above study:

- The boundary layer thickness in case of ramped temperature is always less than isothermal temperature.
- Magnetic parameter 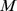
retards the fluid flow.
- Permeability parameter 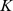 enhances the fluid flow.
- Velocity as well as skin friction decreases due to increasing 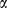.
